# Ghrelin Therapy Decreases Incidents of Intracranial Hemorrhage in Mice after Whole-Body Ionizing Irradiation Combined with Burn Trauma

**DOI:** 10.3390/ijms18081693

**Published:** 2017-08-03

**Authors:** Nikolai V. Gorbunov, Juliann G. Kiang

**Affiliations:** 1 Radiation Combined Injury Program, Armed Forces Radiobiology Research Institute, Uniformed Services University of the Health Sciences, Bethesda, MD 20814, USA; nikolaiv.gorbunov@gmail.com; 2 Department of Pharmacology and Molecular Therapeutics, Uniformed Services University of the Health Sciences, Bethesda, MD 20814, USA; 3 Department of Medicine, Uniformed Services University of the Health Sciences, Bethesda, MD 20814, USA

**Keywords:** animal model, mouse, ghrelin, radiation, burn, brain, hemorrhage, platelet

## Abstract

Nuclear industrial accidents and the detonation of nuclear devices cause a variety of damaging factors which, when their impacts are combined, produce complicated injuries challenging for medical treatment. Thus, trauma following acute ionizing irradiation (IR) can deteriorate the IR-induced secondary reactive metabolic and inflammatory impacts to dose-limiting tissues, such as bone marrow/lymphatic, gastrointestinal tissues, and vascular endothelial tissues, exacerbating the severity of the primary injury and decreasing survival from the exposure. Previously we first reported that ghrelin therapy effectively improved survival by mitigating leukocytopenia, thrombocytopenia, and bone-marrow injury resulting from radiation combined with burn trauma. This study was aimed at investigating whether radiation combined with burn trauma induced the cerebro-vascular impairment and intracranial hemorrhage that could be reversed by ghrelin therapy. When B6D2F1 female mice were exposed to 9.5 Gy Cobalt-60 γ-radiation followed by 15% total skin surface burn, cerebro-vascular impairment and intracranial hemorrhage as well as platelet depletion were observed. Ghrelin treatment after irradiation combined with burn trauma significantly decreased platelet depletion and brain hemorrhage. The results suggest that ghrelin treatment is an effective therapy for ionizing radiation combined with burn trauma.

## 1. Introduction

The adverse effects of ionizing irradiation (IR) to organs and systems have been known since the early 20th century. However, current public health concerns related to radiation exposure are based upon the extensive development and proliferation of nuclear technologies, radiation and nuclear medicine, and nuclear weapon systems, which overall increase the risk of radiation accidents as well as the imminence of nuclear military conflicts. Therefore, it elevates the frighteningly real possibility of mass casualties from terrorist acts with improvised nuclear and radiological devices.

It is widely accepted that irradiation per se at any given dose can affect each biological system. However, there are “limiting” doses, which trigger acute radiation sickness (or acute radiation syndrome, ARS). The acute sickness occurs and propagates due to direct cell lesions and cascades of indirect reactive responses that can ultimately lead to the impairment of sensitive tissues sustaining crucial immunochemical and metabolic homeostasis, breach of biological barriers, post-irradiation sepsis and multiple organ failure (MOF) [1,2,3,4,5,6,7,8]. The hematopoietic form of acute radiation sickness or syndrome (H-ARS) is commonly developed upon total body irradiation (TBI) and is so far considered the only “curable form” of acute radiation injury [2,9]. It has been well documented that the pathogenesis of a H-ARS disease is featured by the depletion of the peripheral white blood cells (WBCs), a reduction in responses to growth factors and hormones, and the suppression of the clonogenic potential of lymphoid, mucosal, endothelial and epithelial tissues. These effects can ultimately lead to the attrition of associated tissue barriers and the development of numerous reactive and metabolic responses. Among them, the following constitute high risk to MOF: (i) hypercytokinemia and non-septic inflammation; (ii) neurogenic, metabolic, oxidative, and electrophilic stress; (iii) coagulopathy; (iv) enteropathy; (v) bacteremia and sepsis; and (vi) fluid loss, electrolyte imbalance, and interstitial hemorrhage [2,9,10]. All together these alterations are considered to be major factors of MOF and moribundity in H-ARS. It is noted that damage to microvascular networks and the suppression of the clonogenic potentials of bone marrow—a major source of endothelial progenitors—constitutes one of the most important IR-impacts on parenchymal tissues which otherwise display relatively high radio resistance to H-ARS-induced radiation doses (e.g., brain, lung and heart parenchyma) [5,6,8,11].

The emerging threat from acute radiation exposure evolves a need for the development of new countermeasures and modalities effective in response to and management of radiation injury [12]. In recent triage for the management of nuclear/radiological accidents, it is suggested that a scenario of casualties from radiation exposure alone (i.e., in the absence of other injuries) is not realistic. Thus, it has become abundantly clear that radiation exposure combined with many kinds of other injuries, including burns—i.e., radiation combined injuries (CI)—often results in a negative synergistic response more harmful than the sum of the individual injuries. Animal studies clearly demonstrate that hemorrhage [13], wounds [14,15,16], sepsis [17], or burns [16,18,19] exacerbate ARS. Observations of personnel exposed to IR also indicate that burns complicate the morbidity and mortality of the sickness [20]. On the other hand, it became evident that IR-induced suppression of progenitor cells in wounded tissues and bone marrow complicates tissue renewal, neovascularization in trauma healing, and the remodeling of microvascular beds affected by IR and post-IR reactions [21,22]. Therefore, it is essential to develop and test prospective countermeasure agents or combinations thereof for the purpose of CI management. With this perspective, we previously investigated the beneficial effect of ghrelin, a growth-hormone-releasing peptide, in mitigating H-ARS and boosting recovery from CI-associated trauma in mice [23]. The reported data indicated that the administration of ghrelin to CI mice can increase their survival, mitigate body-weight loss, accelerate wound healing, and increase hematocrit values and the number of white blood cells (namely neutrophils and lymphocytes) and platelets, as well as mitigating the CI-induced depletion of hematopoietic cells in the bone marrow [23]. Seemingly, these results provide evidence that ghrelin therapy can improve CI-survival and recovery via (at least in part) ameliorating the damage to lymphoid tissues and attenuating the effects of leukocytopenia and thrombocytopenia [23]. In the present report we hypothesized that ghrelin administration can prevent CI-induced cerebro-vascular impairment and intracranial hemorrhage and by these means can contribute to the amelioration of morbidity and mortality in sequelae of the illness. The rationale is that (i) firstly, the brain is one of the most highly perfused organs in the body and therefore, along with lung, has the most extensive microvascular network; (ii) secondly, numerous data suggest that while IR-induced endothelial apoptosis can “directly” disturb the blood–brain barrier, the vascular reactive response to the delayed bacteremia and sepsis can provoke intraparenchymal hemorrhage; (iii) thirdly, while ghrelin is defined as a gastrointestinal hormone that is primarily synthesized and secreted by the stomach, ghrelin is also expressed in the endothelial cells of blood vessels [24,25,26,27,28]; and (iv) fourthly, *in vivo* ghrelin production is shown to be dramatically affected by irradiation and CI [17]. Thus, so far along with its other physiological effects such as (i) stimulation of growth hormone release from the anterior pituitary gland and hypothalamus; (ii) induction of a positive energy balance; (iii) stimulation of neurogenesis and neuroprotection against damage due to ischemia, traumatic brain injury and neuromediator excitotoxicity; and (iv) suppression of sepsis and protection of vascular endothelium against septic inflammation, ghrelin has been also demonstrated to sustain endothelial function and angiogenesis [17,24,25,26,27,28].

The data presented in this report demonstrate a decrease in incidents of intracranial hemorrhage in CI animals treated with ghrelin compared to CI animals treated with vehicle that sustains the main hypothesis.

## 2. Results

### 2.1. Brain Hemorrhagic Lesions in the Radiation Combined Injuries (CI) Model

**Figure 1:**
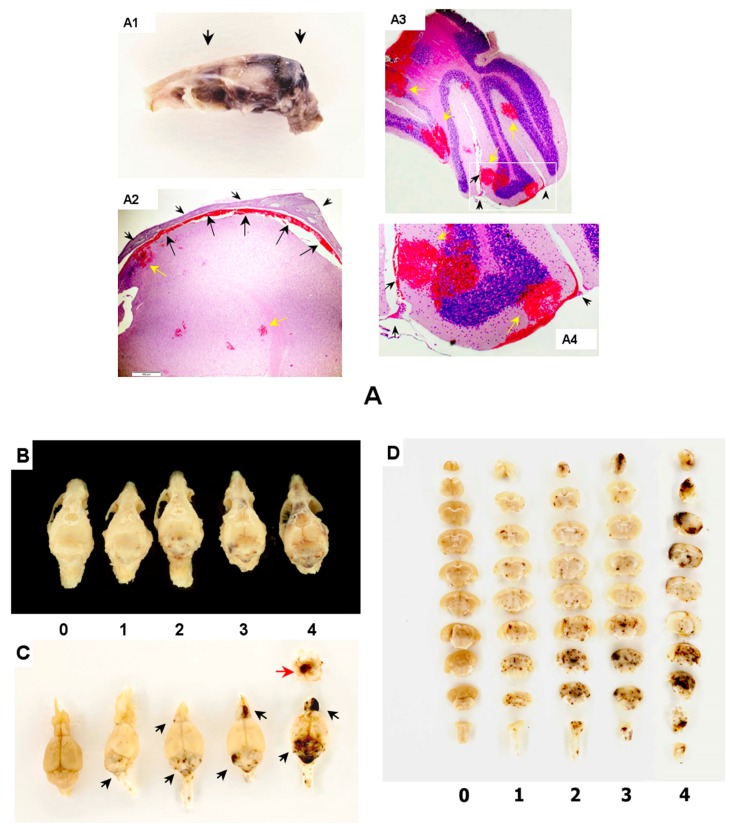
Brain histology and gross-pathology after irradiation combined with burn trauma (CI). (**A**) Intracranial hemorrhage in brain of animals exposed to radiation combined with burn trauma (CI; moribund animal at day 16 post-CI); (**A1**) Midsagittal projection of brain in skull: hemorrhagic surface appears in dark brown color. Black arrows indicate sites of the coronal sections shown in (**A2**–**A4**) with the histology of the coronal sections of the brain; H&E staining for extravasated red blood cells; (**A2**) Histology of subdural and intraparenchymal hemorrhage (indicated with black and yellow arrows respectively) in a selected area of the cortex (i.e., in the motor and frontal cortex). The cranium is indicated with black arrow heads; (**A3**) Histology of the subarachnoid and intraparenchymal hemorrhage (indicated with black arrow heads and yellow arrows respectively) in a selected area of the cerebellar cortex (i.e., in paramedian lobule, copula pyramis, crus cerebellum 2); (**A4**) Magnification of a selected area shown in (**A3**) (white rectangle); (**B**) Representative images of skulls from dorsal projections of intracranial hemorrhages; (**C**) Respective images of brains collected at end-point of CI-observations; Extra axial (subdural) hemorrhage is indicated with red arrow; intra-axial (intra parenchymal) hemorrhage is indicated with black arrows. The collected skulls and brains were positioned in the pecking order of the sizes/extents of the brain hemorrhagic surfaces in a scale from “0” through “4” (panels **B**,**C**); (**D**) The presented sets of coronal sections were positioned in the pecking order of the sizes/extents of the hemorrhagic surfaces of the sections in a scale from “0” through “4”.

Gross pathology assessments of skulls and brains obtained from B6D2F1/J mice revealed that all moribund animals subjected to CI experienced intracranial hemorrhages that varied in the extent, grade, hemorrhage type, and depth of lesions. The scope of intra-axial and extra-axial lesions included (i) intra-parenchymal hemorrhage; (ii) subdural hemorrhage; (iii) subarachnoid hemorrhage; and (iv) hemorrhage to the cranial and the mandibular bones of the skulls (Figure 1). Projections of hemorrhagic zones in skulls appeared in dark brown (Figure 1(A1)). The presence of subdural and subarachnoid hemorrhage was determined with histopathology analysis (Figure 1(A2–4)). With the indicated variations of lesions in the collected specimens, the index of hemorrhage severity (IHS) was arbitrary denominated as follows:

Level 1—Characterized by the appearance of isolated intra-parenchymal and subarachnoid hemorrhage in a form of hemorrhagic petechiae observed predominantly in cerebellum, medulla oblongata, pons, midbrain, cerebral cortex, and corpus callosum. The surface of the hemorrhagic lesions constituted less than 1% of the total surface of the brain.

Level 2—Characterized by the presence of numerous hemorrhagic petechiae observed predominantly in cerebellum, medulla oblongata, pons, the midbrain, cerebral cortex, corpus callosum, striatum, thalamus, hypothalamus, and the olfactory bulb. The surface of the hemorrhagic lesions constituted 1% to 3% of the total surface of the brain.

Level 3—Characterized by the presence of subarachnoid purpural hemorrhage and deep intra-parenchymal purpural hemorrhage observed predominantly in the hindbrain, the midbrain and the olfactory bulb; the presence of numerous hemorrhagic petechiae in the entire structures of the telencephalon and the diencephalon was determined. The surface of the hemorrhagic lesions constituted 3% to 10% of the total surface of the brain.

Level 4—Characterized by the development of deep ecchymotic/confluent intra-parenchymal hemorrhage that occurred in the hindbrain, midbrain, and the entire structures of the telencephalon and diencephalon. Moreover, the presence of confluent hemorrhagic lesions in the subdural and subarachnoid area, pia matter, as well as a presence of intraventricular hemorrhage and hemorrhage to the cranial and the mandibular bones of the skull were observed. The surface of the hemorrhagic lesions constituted over 10% of the total surface of the brain.

Representative images of the dorsal projections of the skulls and brains with extra-axial and intra-axial hemorrhage are shown in Figure 1B,C, where the specimens are positioned in the pecking order of IHS levels defined above. Representative images of the coronal sections of the brains are shown in Figure 1D. Assessment of the coronal sections revealed the depth of the hemorrhagic lesions that contributed to IHS. This “surface-based” assessment of intracranial hemorrhage (Figure 1B,C) was correlated with the observation of the brain coronal sections showing the depth of intra-axial hemorrhages (Figure 1D).

### 2.2. Effect of Ghrelin Administration on Incidents of Hemorrhagic Lesions and the Ratio of Index of Hemorrhage Severity (IHS) in the CI Model

**Figure 2:**
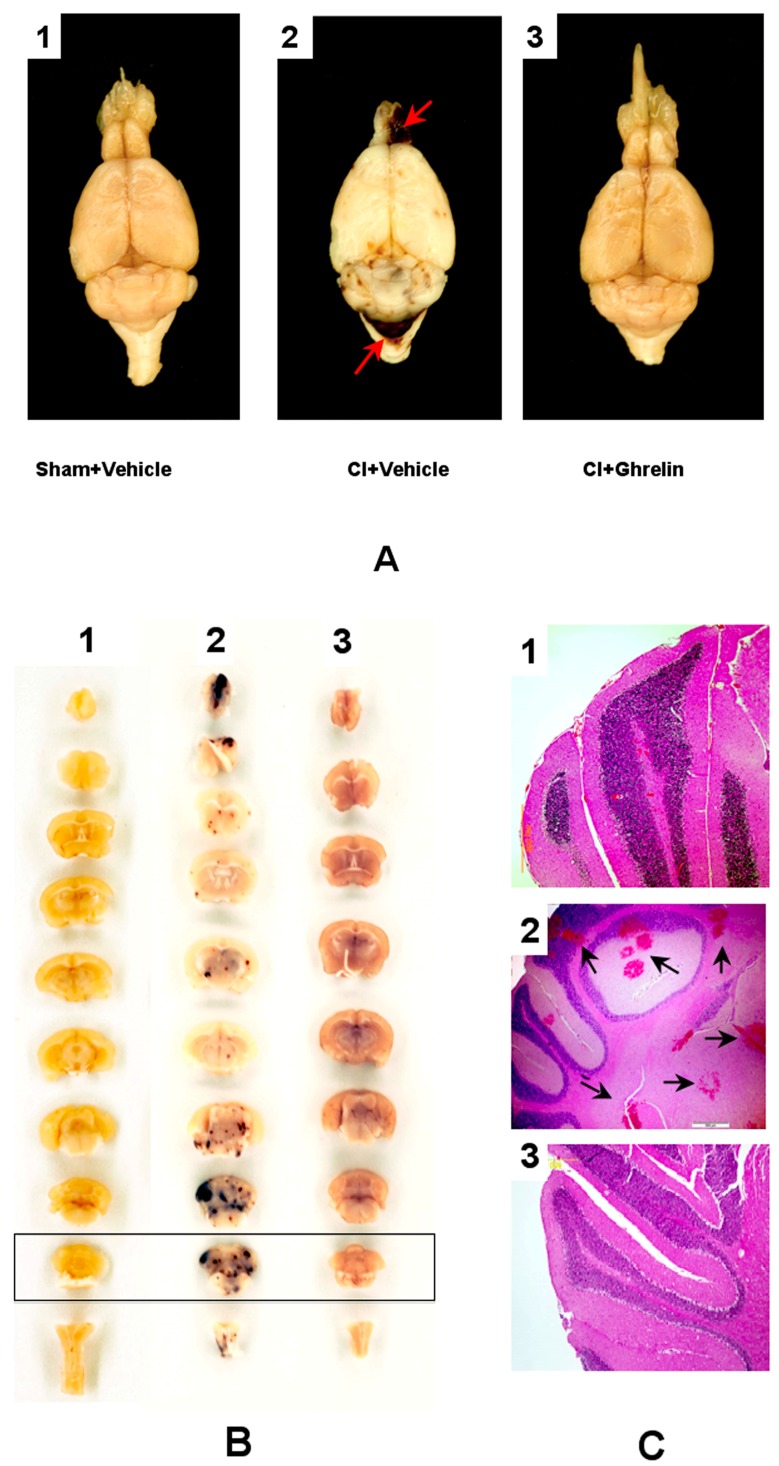
Gross pathology of intra-axial (intracerebral) hemorrhage of brain in a CI-vehicle animal compared to sham and CI-ghrelin animals (**A**) with red arrows indicating blood extravasation (**A2**). (**B**). Respective coronal sections of the brains; (**C**). Histopathology of selected sections of cerebellum specimens with H&E staining. 1—Sham; 2—Moribund (Vehicle) animal at the 13th day post-CI; Blood extravasation is indicated with black arrows; 3—A specimen from an animal (CI + Ghrelin cohort) at the 30th day post-CI.

All the previously published data indicated that ghrelin therapy for CI significantly mitigated hematopoietic injury and decreased the mortality rate of the animals [23]. The purpose of the current report is to demonstrate that the observed “pro-survival effects” of ghrelin were correlated with decreases in the incidents of intracranial hemorrhage in B6D2F1/J mice, namely, with the improvement of CI-related cerebro-vascular impairment. Indeed, the further conducted gross pathology assessment of the brain specimens indicated that intracranial hemorrhagic lesions of different extents were present in all dead mice. Meanwhile, there were no signs of hemorrhagic lesions in the CI-surviving mice at the end of experiment. Representative gross-pathology images of intra-axial (intracerebral) hemorrhage in dead CI vehicle-treated animals compared to sham and CI ghrelin-treated animals are shown in Figure 2A. The images of the respective cranial sections and microsections (H&E staining) of selected parts of the cerebellum specimens are presented in Figure 2B,C. As appeared, massive deep confluent lesions occurred predominantly in the hindbrain, cerebellum, brain base, and the olfactory bulbs that were accompanied by subarachnoid hemorrhage in the structures (Figure 2A,B). The surface of the hemorrhagic lesions constituted about 10% of the total surface of the brain. Moreover, numerous petechiae occurred in the frontal cortex, striatum, hypothalamus, visual cortex, pons and medulla oblongata. The presence of interstitial hemoglobin in the hemorrhagic lesions was documented with histopathology (H&E staining followed by light microscopy) (Figure 2C). Overall, the IHS for the presented brain specimen was defined as a score of three.

**Figure 3:**
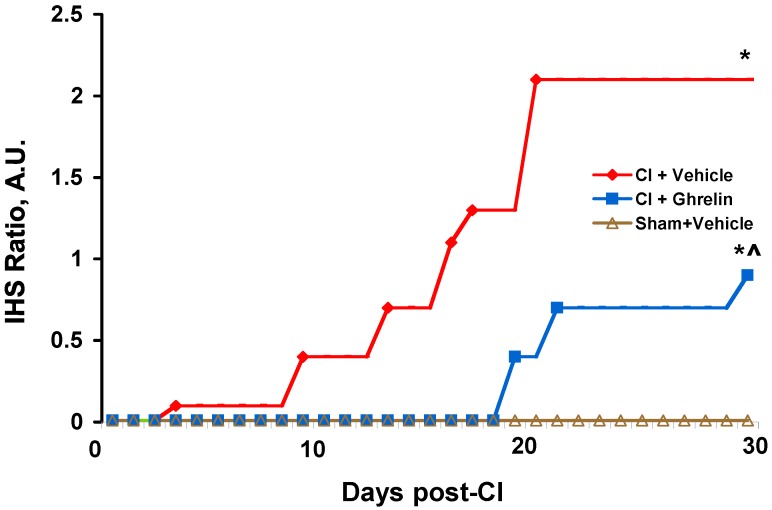
Histogram representing ratio of the IHS defined for the CI-experimental groups over a 30-days observation period. * *p* < 0.05 vs. sham + vehicle group; ^ *p* < 0.05 vs. CI + vehicle group.

Data obtained from the gross pathology and analysis of the daily tally of IHS-ratios for the CI animal cohorts were plotted and are presented in Figure 3. As shown in the histogram, the defined ratios for the IHS were significantly lower in the CI + ghrelin cohort compared to the CI + vehicle cohort.

### 2.3. Ghrelin Administration Mitigates Platelet Depletion Caused by Radiation Combined with Burn Trauma (CI)

**Figure 4:**
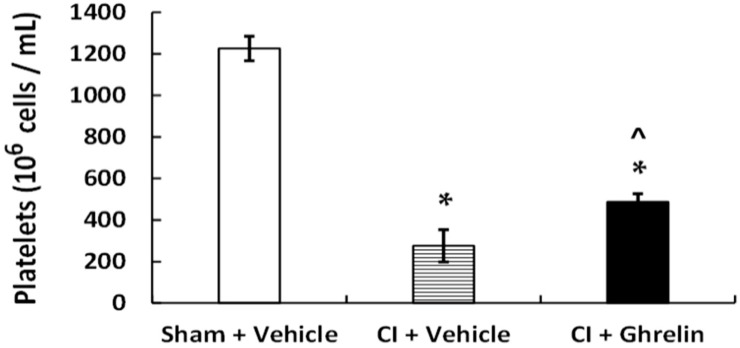
Treatment with ghrelin after radiation combined with burn trauma significantly mitigates platelet depletion. Animals were treated with ghrelin or vehicle after irradiation followed by burns (CI). Platelets were counted in surviving CI animals. *n* = 6–10 per group. * *p* < 0.05 vs. sham + vehicle group; ^ *p* < 0.05 vs. CI + vehicle group.

CI is known to induce platelet depletion [23]. As shown in Figure 4, in surviving CI mice, platelets were counted and indeed platelet depletion was observed (in 10^6^ cells·mL^−1^; sham + vehicle: 1226 ± 59 vs. CI + vehicle: 276 ± 90; *p* < 0.05). Treatment with ghrelin mitigated this depletion and significantly elevated platelet counts back to 487 ± 45 × 10^6^ cells·mL^−1^ (*p* < 0.05).

## 3. Discussion

The intention of this report is to demonstrate that the acute sickness and moribundity that developed in the B6D2F1/J mice due to CI were associated with intracranial hemorrhage. Administration of ghrelin injected sequentially three times (i.e., at 24, 48, and 72 h) after CI mitigated sickness, moribundity and incidents of intracranial hemorrhage. To our knowledge, this is the first report indicating the mitigative effect of ghrelin against microvascular injury in brain of B6D2F1/J mice subjected to IR combined with skin burn trauma.

It is well documented that damage to the microvascular networks constitutes one of the most important outcomes in the pathogenesis of sickness due to total or partial body IR [5,6,21,29]. The resulting circulatory effects of acute IR are often complicated by a massive release of numerous reactive factors—coagulopathy, suppression of vascular growth factors, and vascular remodeling. Thus, it can ultimately affect peripheral perfusion [30,31]. It should be noted that the microvascular histohematic barriers are arranged by vascular endothelial cells which, along with the basement membrane and perocytes, sustain circulatory homeostasis. Interestingly, while the kinetics of radiation damage to endothelial cells means that they do not count among the exceptionally radiosensitive phenotypes (e.g., lymphocytes or bone marrow progenitor cells), delayed pro-inflammatory reactions, protracted cytotoxic stress, and bacteremia can provoke permeability of the endothelial layer and of the basement membrane to fluids and blood cells leading to changes in the extracellular milieu ultimately reflected in a loss of parenchymal cells and parenchymal necrosis [8,11,21,30,31]. Thus, it has become apparent over recent years that IR-induced microvascular impairment contributes to both acute and delayed post-IR effects in normal tissue. Among them interstitial hemorrhage, hypoxia, and necrosis are life-threatening outcomes, which not only represents a great challenge for the development of countermeasures against radiological/nuclear accidents but also can complicate outcomes in radiation therapy [5,21,32,33].

The existing data on CI models indicate that the administration of ghrelin to CI rats and mice can increase their survival seemingly due to the mitigation of bone marrow injury and the suppression of neuronal systemic inflammatory effects [17,23]. Thus, it has been shown previously that ghrelin therapy can sustain hematopoietic recovery in B6D2F1/J mice subjected to irradiation combined with burns [23]. While using another model, i.e., radiation injury combined with severe sepsis in rats, ghrelin can interfere with the release of norepinephrine from the stimulated gut sympathetic fibers and, by this means, can induce a down-regulation of the pro-inflammatory cytokines (IL-1β, IL-6, and TNF-α) activated by norepinephrine [17]. In another recent paper Jacob and co-authors have suggested that the anti-inflammatory responses associated with either ghrelin up-regulation or ghrelin administration are initiated by stimulation of the cholinergic parasympathetic pathways in the dorsal vagal complex via stimulation of GHSR-1a receptor, a ghrelin molecular counterpart [34]. These authors proposed that radiation injury combined with severe sepsis could unbalance sympathetic/parasympathetic nervous systems, ultimately leading to adverse systemic responses [34]. Under these circumstances, ghrelin’s beneficial effects could be due to a rebalancing of misregulated sympathetic/parasympathetic pathways [34]. In conjunction with data reported in the current paper we would expect that ghrelin administration could also mitigate the neuro-inflammatory events activated following brain hemorrhage–neuroinflammation, which is a challenge to diagnose and manage [35]. The exact role of ghrelin in the brain inflammation process and on coagulation systems remains to be unfolded.

Our observations indicated that the development of intracranial hemorrhage in the B6D2F1/J strain occurred at one or another grade in all cases of moribundity and animal death. The role of hemorrhagic lesions in the pathogenesis of the disease and the mortality rate requires further investigation. However, it would be reasonable to assume that the administration of ghrelin can mitigate the thrombopoietic effects of CI [23]; therefore, it would be possible that ghrelin-mediated improvement of platelet counts can promote the stopping of the brain hemorrhage after CI. Moreover, it is evident that radiation combined with burn trauma increases miR-690 and miR-223 in serum [36]. Likewise, radiation combined with hemorrhage increases let-7e, miR-30e, and miR-29b [13]; radiation combined with wound increases miR-34a [Kiang et al., unpublished data]. The possibility that ghrelin treatment modifies microRNA that are associated with thrombopoiesis cannot be excluded and needs to be explored.

To address the growing demand for R&D of prospective remedies for the management of vascular brain injury, we have proposed a mouse model (B6D2F1/J mice) of CI-induced intracranial hemorrhage and tested the influence of ghrelin on incidents of brain hemorrhagic lesions. The results from our experiments indicate that besides the previously reported systemically beneficial effects and improvement of wound healing [17,23,34], the administration of ghrelin can also ameliorate neurovascular recovery from CI.

## 4. Materials and Methods

### 4.1. Experimental Design

B6D2F1/J female mice were randomly divided into three groups: (1) sham + vehicle, (2) CI + vehicle, and (3) CI + ghrelin. Each group received topical gentamicin cream and were administered with oral levofloxacin. Survival experiments were repeated one time with *n* = 10–11 mice per group.

### 4.2. Animals

B6D2F1/J female mice at 12–20 weeks of age were used because male mice are aggressive and will chew each other resulting in unnecessary physical injury that is beyond the wound injury described in the experimental protocol (approved by the AFRRI Institutional Animal Care and Use Committee, identification code: 2010-12-016; dated 11 February 2011). The female mice were obtained from the Jackson Laboratory (Bar Harbor, ME, USA). They were sent to AFRRI and kept in plastic microisolator cages bedded with hardwood chip. They received commercial rodent chow and acidified tap water *ad libitum*. They were acclimated about eight days before use for experiments. The animal holding rooms had a temperature of 21 °C, 10% relative humidity, and 12 h lighting cycles (6 AM light to 6 PM dark).

### 4.3. Gamma Irradiation

The procedure of irradiation has been described previously [23]. Briefly, mice were exposed to Cobalt-60 γ-photon radiation using Panoramic Cobalt-60 irradiator (9.5 Gy, whole-body, bilateral, 0.4 Gy·min^−1^), with the field uniformed within ±2%. The mapping data determined each radiation exposure time. The cobalt-60 decay was corrected. Differences in the mass energy absorption coefficients for soft tissues and water were taken into consideration. An ionization chamber adjacent to the mouse rack was used to verify the accuracy of the actual dose delivered to the animals. The verification of dose was calculated aiming the midline soft tissue of animals.

### 4.4. Skin Injury

The procedure of skin burn has been described previously [23]. Briefly, the dorsal surface of mice was shaved. Within 1 h after irradiation or sham-radiation, these animals were anesthetized by inhaling methoxyflurane. Then, each animal was placed on a platform. Using a 1 × 1-inch custom designed template positioned centrally over the shaved dorsal skin surface, a 15% total-body-surface skin burn was carried out. A volume of 0.25 mL of 95% ethanol was applied evenly to the dorsal skin surface, which was exposed by the template. The ethanol was ignited and allowed to burn for 12 s [16,37]. All mice subjected to the skin injury were given 0.5 mL sterile 0.9% NaCl intraperitoneally (i.p.), which contained 150 mg·kg^−1^ of acetaminophen (AmerisourceBergen, Glen Alen, VA, USA) and 0.05 mg·kg^−1^ of buprenorphine immediately after skin injury to alleviate pain. Four hours later, mice were given a second dose of 150 mg·kg^−1^ of acetaminophen. Skin-wounded only mice received only one dose of 150 mg·kg^−1^ of acetaminophen immediately after skin injury; sham-operated mice only received one i.p. injection of 0.5 mL sterile 0.9% NaCl.

### 4.5. Ghrelin Treatment

Ghrelin was purchased from Phoenix Pharmaceutical (Burlingame, CA). Three doses of 113 μg·kg^−1^ were administered by lateral tail-vein injections [23] in a volume of 0.2 mL 24, 48 and 72 h after CI. The vehicle given to control mice was sterile 0.9% sodium chloride solution for injection, USP.

### 4.6. Antimicrobial Agents

Gentamicin sulfate cream, 0.1% (generic, E. Fougera and Co., Melville, NY, USA, NDC 0168-007-15), was applied daily for 10 days to the skin injured area on days 1–10. Levofloxacin (LVX), (generic, Aurobindo Pharma, Ltd., Mahaboob Nagar, India, NDC 65862-537-50), 100 mg·kg^−1^ in 0.2 mL per mouse, was administered *per os* (p.o.) daily for 14 days on days 3–16. Briefly, a 500 mg tablet was crushed by mortar and pestle. The LVX in the powder was dissolved in a volume of sterile water approximately one-third of the total volume required to prepare the concentration needed for the average body mass of the mice to be treated. The mortar was rinsed with the remaining two-thirds volume of sterile water. The combined suspension was centrifuged to remove the particulate filler and the supernatant solution was passed through a 0.45 μm membrane filter into a sterile amber bottle, which was sealed with a sterile rubber stopper and stored at 4–8 °C.

### 4.7. Score of Intracranial Hemorrhagic Lesions using Index of Hemorrhage Severity (IHS)

Entire mouse skulls were collected at the end-point of observations, preserved in 10% neutral buffered formalin, and then the brains were extracted from the cranial cavities and subjected to routine gross pathology. The severity of intracranial hemorrhage (intra-axial and extra-axial) was scored with the provision of the scoring system for hemorrhage assessment previously introduced by Yelverton [38]. In brief, firstly the skull and brain regions with hemorrhage were assessed for the extent of lesions on the basis of the number of the intra-axial and extra-axial structures affected by hemorrhage (i.e., cerebellum, pons, medulla oblongata, the structures of telencephalon and diencephalons, bones, subdura, pia). Then, we defined the grade of lesions as a scope (or percentage) of surface area (including base of the brain) with extravasated blood. A presence of intense color contrast between the intact brain and bone tissues (i.e., white-pinkish color) and extravasated blood (i.e., dark-brown color) allowed the definition of the scope and edge of hemorrhagic lesions that was further confirmed with histopathology analysis (see below). Morphometric data for structures of mouse brain were obtained from the report by Badea et al. [39]. Hemorrhage type was introduced to the qualitative nature of a hemorrhagic lesion (i.e., either petechia or purpura or ecchymosis or confluent hemorrhage). The depth of the hemorrhagic lesions was determined on the basis of the degree of penetration, i.e., subdural hemorrhage, hemorrhage to pia (subarachnoid), deep parenchymal and intraventricular hemorrhage. Thus, the index of hemorrhage severity (IHS) for intracranial hemorrhage was determined as a sum of extent, grade, severity type, and lesion depth scores; where “0” was assigned to the absence of intra-axial and extra-axial extravasation; while “4”—the maximum number—was assigned to the largest appearance of hemorrhagic lesions characterized by: deep ecchymotic/confluent intraparenchymal hemorrhage in the entire structures of the telencephalon and diencephalons along with the presence of large confluent hemorrhagic lesions in the subdural and subarachnoid areas, pia matter, as well as the presence of hemorrhage to the cranial and the mandibular bones of the skull.

The IHS-ratio for each CI-experimental group at each observation day was then calculated as follows: cumulative IHS per group (i.e., a sum of IHS obtained from each individual moribund animal in a group to the point of each tally day) was divided by 10 (i.e., by the number of animals in each group at start-point, day “0”). The calculated data were presented in a histogram of IHS-ratio versus observation days.

### 4.8. Histology

Intact mouse brains fixed in 10% neutral buffered formalin were obtained as described in the previous paragraph. The brains were subjected coronal sectioning as shown in Figure 1 and Figure 2. The scopes of intraparenchymal hemorrhagic lesions in the sections were graded on a semi-quantitative scale of 0 (absence of hemorrhage) to 4 (severe hemorrhage). Then, the obtained tissue specimens were embedded in paraffin, cut into 5 μm sections, stained with hematoxylin and eosin, and examined by light microscopy for the presence of extravasated red blood cells in the parenchymal tissue and subdural and/or subarachnoid space (Figure 1 and Figure 2).

### 4.9. Measurements of Platelet Counts

Blood samples collected in EDTA tubes were assessed with the ADVIA 2120 Hematology System (Siemens, Deerfield, IL, USA). Platelet counts were expressed as mean ± SEM. with units of 10^6^ cells·mL^−1^.

### 4.10. Statistical Analysis

Parametric data were expressed as the mean ± SEM. For each survival experiment, 20–22 mice per group were tested on an individual basis. IHS-ratios for CI-vehicle and CI-ghrelin animal groups over 30 days of observation were compared using the one-way ANOVA and Tukey post hoc test. Platelet data were analyzed with Student’s t-test. A significant level was at or less than 5%.

## 5. Conclusions

In conclusion, we show that ionizing radiation followed by skin surface burns induces cerebro-vascular impairment and intracranial hemorrhage as well as platelet depletion. The results suggest that platelet depletion probably partly contributes to intracranial hemorrhage, thus this intracranial hemorrhage partly leads to the final mortality. Treatment with ghrelin after irradiation combined with burn trauma significantly decreased platelet depletion and brain hemorrhage. To clarify the role of ghrelin in mitigating the CI-induced brain inflammation process and coagulation systems requires further exploration. However, taken together, the results presented in this report and the data in literature show that ghrelin treatment is an effective therapy for combined radiation injury.

## Abbreviations

ARS	Acute radiation syndrome
CI	Radiation combined injury
H-ARS	Hematopoiesis-acute radiation syndrome
IR	Ionizing radiation
IHS	Index of hemorrhage severity
i.p.	Intraperitoneally
p.o.	*per os*
